# Could higher hospital spending improve survival in patients with esophageal squamous cell carcinoma? A multicenter retrospective cohort study

**DOI:** 10.3389/fonc.2025.1668017

**Published:** 2026-01-22

**Authors:** Lei Chen, Wei Yang, Lei Chen, Ruiping Xu, Wenlei Yang, Fangfang Liu, Yu He, Zhen Liu, Bolin Hou, Liqun Zhang, Miaoping Lin, Yaqi Pan, Zhonghu He, Yang Ke

**Affiliations:** 1 Key Laboratory of Carcinogenesis and Translational Research (Ministry of Education/Beijing), Department of Genetics, Peking University Cancer Hospital & Institute, Beijing, China; 2 Cancer Hospital of Shantou University Medical College, Shantou, China; 3 Anyang Cancer Hospital, Anyang, China; 4 Chinese Preventive Medicine Association, Beijing, China; 5 Linkdoc AI Research (LAIR), Beijing, China; 6 State Key Laboratory of Molecular Oncology, Beijing Key Laboratory of Carcinogenesis and Translational Research, Department of Genetics, Peking University Cancer Hospital & Institute, Beijing, China

**Keywords:** cohort study, esophageal squamous cell carcinoma, hospital spending, multicenter, overall survival

## Abstract

**Background:**

The hospital spending of patients with esophageal squamous cell carcinoma (ESCC) have been increasing over years, imposing a heavy economic burden on these patients. However, little is known about the association between spending and their overall survival (OS).

**Methods:**

We recruited 11,037 ESCC patients who were admitted between August, 2009 and December, 2018 at the Southern Center (Cancer Hospital of Shantou University Medical College), and between January, 2012 to December, 2017 at the Northern Center (Anyang Cancer Hospital). Spending terciles were the exposure measure, and OS was the outcome. OS in terciles 2 and 3 was compared with OS in tercile 1 (the lowest spending tercile) using Cox regression models. Analyses were stratified by TNM stage and study center.

**Results:**

Monthly hospital spending followed an “L-shaped” trend. After a maximum follow-up of 12.52 years, the median survival time was 4.70 years. Higher spending was associated with worse OS in stage 0-II patients (adjusted HR_tercile 3 vs 1_ = 1.55, 95% CI: 1.27-1.89), but with better OS in stage III-IV patients (adjusted HR_tercile 2 vs 1_ = 0.82, 95% CI: 0.74-0.90; adjusted HR_tercile 3 vs 1_ = 0.73, 95% CI: 0.64-0.83). These associations were consistent across both the Southern and Northern Centers.

**Conclusions:**

The findings suggest that early-stage ESCC patients may benefit from more conservative treatment approaches, whereas advanced-stage patients require comprehensive and sufficient treatment.

## Graphical Abstract

**Figure:**
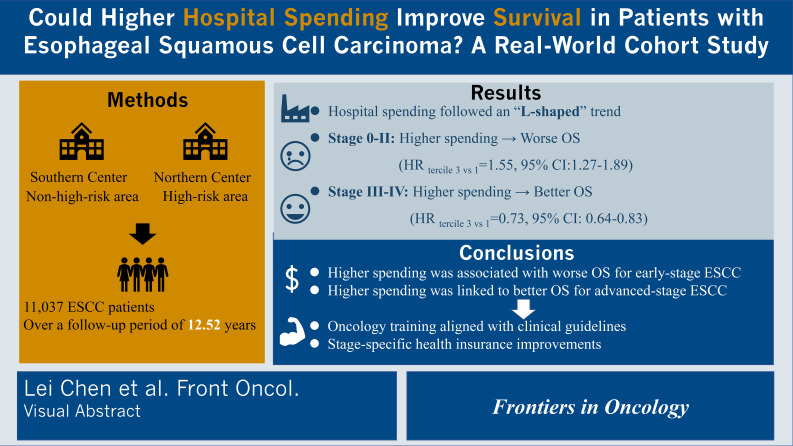

## 1 Introduction

Globally, esophageal cancer (EC) is the seventh most common cancer and the sixth leading cause of cancer-related deaths (1). The two major subtypes of EC are esophageal squamous cell carcinoma (ESCC) and esophageal adenocarcinoma (EAC). ESCC accounts for 85.8% of all EC cases and is particularly prevalent in Eastern Africa and Eastern Asia, with the highest incidence rates observed in China (1, 2). The prognosis of ESCC diagnosed at a symptomatic stage is usually fatal, with a 5-year survival rate of only 27.9% in China (3). The main reason for the low survival rate is that most patients are asymptomatic and undetected until they are at advanced stage. Treatment for advanced-stage EC is costly and places a significant economic burden on patients, particularly in rural high-risk areas (4), where ESCC treatment tended to be more expensive than EAC (5). Medical expenses for EC patients have been steadily increasing (5–7), with the average total cost per hospitalization now equivalent to an entire year’s gross domestic product per capita (8).

Given the severe disease and economic burdens caused by EC (6), improving care and reducing costs for EC patients have become increasingly recognized priorities (9, 10). Unfortunately, there have been no high-quality, large-scale studies on the association between spending and overall survival (OS) in ESCC. Existing research has primarily focused on the cost-effectiveness of different therapies, often using models targeting advanced-stage patients. One study found that higher spending was associated with better 3-year OS for 232 ESCC patients who underwent neoadjuvant chemoradiotherapy (11). Other modeling studies have shown that combining specific anticancer drugs with chemotherapy is much more expensive, yielding only slightly better OS compared to chemotherapy alone (12). For other types of cancer, studies have produced mixed results (13–17). To date, only one relevant study has examined ESCC patients undergoing neoadjuvant therapy (11), and little is known about the relationship between spending and OS in ESCC, especially for early-stage patients. Therefore, evaluating the impact of hospital spending on OS for ESCC patients using large-scale real-world data is essential, particularly in resource-limited areas.

To address this gap, we conducted a multi-center real-world study to evaluate the association between hospital spending and OS in ESCC patients residing in both high-risk and non-high-risk regions for ESCC, aiming to provide high-quality evidence to facilitate informed clinical decision-making and policy development for ESCC. This analysis is situated within the context of specialized oncology care in China. Accordingly, we have defined our cost evaluation from the standpoint of the specialized cancer hospital, focusing on “first specialized treatment spending.” This encompasses all costs from a patient’s first admission for treatment, which, under the standardized referral pathways, occurs after initial diagnosis and staging are typically completed in tertiary general hospitals or regional screening programs.

## 2 Methods

### 2.1 Study design and patients

We conducted this real-world study at two centers, the Southern Center (Cancer Hospital of Shantou University Medical College, Shantou City, Guangdong Province, China) and the Northern Center (Anyang Cancer Hospital, Anyang City, Henan Province, China), as published elsewhere (18). Located in the southeast coastal area, Shantou city has an EC incidence rate (age-standardized: 11.43 per 100,000 person-years) comparable to the national average (19). Anyang City is located in the Taihang Mountain region, which is a high-risk area for EC with an incidence rate up to five times the national average level (19, 20). Both centers are the only Grade-A tertiary cancer specialized hospitals in their respective localities, offering comprehensive cancer treatment services within their catchment regions.

We consecutively recruited patients diagnosed with ESCC who were first admitted to hospital between August 1, 2009 and December 31, 2018 at the Southern Center, and between January 1, 2012 and December 31, 2017 at the Northern Center. The follow-up at the two centers was up to June 7, 2022 and August 23, 2022, respectively. We excluded patients who 1) didn’t receive anticancer treatment; 2) had missing basic information or cost data; 3) had a follow-up period of less than 6 months. (**Supplementary Figure S1**).

### 2.2 Data collection and processing

The electronic clinical records of ESCC patients (N = 11037) were extracted from the Hospital Information System. Details of information collection and data quality control are provided in the **Supplementary Materials**. Missing values for continuous variables were imputed with medians and an “Unknown” group was created to account for missing categorical variables.

### 2.3 Cost calculation

**Figure 1:**
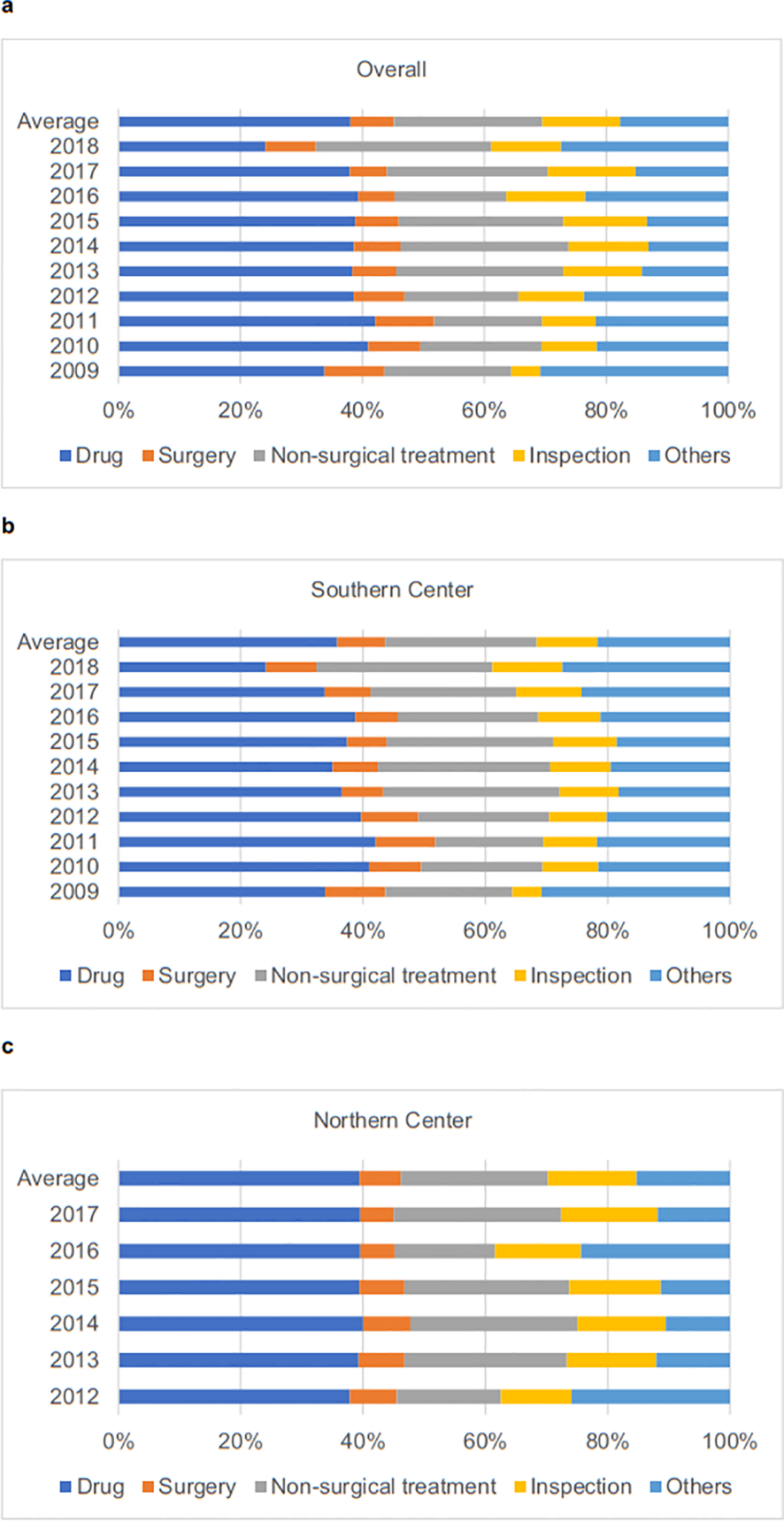
The proportional breakdown of hospital spending for ESCC patients. [**(A)** Overall; **(B)** Southern Center; **(C)** Northern Center]. Non-surgical treatment includes radiation and oxygen therapy. Others include nursing, bed, laboratory test and diagnosis. All costs were reported in US Dollars based on the 2023 value. ESCC, esophageal squamous cell carcinoma.

Hospital spending including the costs for drugs, surgery, non-surgical treatment, inspection, and others, was exported by the hospital electronic medical record system. Non-surgical treatment included radiation and oxygen therapy. Others included nursing, bed, laboratory test and diagnosis. These costs represent the total hospital spending over the entire treatment period for ESCC, from the first to the last treatment within the same hospital (**Figure 1**). The mean hospital spending was calculated as the total hospital spending per patients. All costs were reported in Chinese Yuan (CNY) based on the 2023 value, which were inflated using the year-specific personal health care consumer price index (CPI) of Southern Center (Shantou city) and Northern Center (Anyang city), respectively (**Supplementary Table S1**). The costs were then converted into US Dollars using the 2023 purchasing power parity exchange rate ($1 = ¥3.64) (21). In addition, two sensitivity analysis were performed. The first accounted for total costs from an indirect perspective, including hospital spending and time costs, as detailed in the **Supplementary Materials**. The second addressed a potential concern: survival time could influence spending group assignment, making it difficult to determine if higher spending improves survival or if longer survival simply allows more spending. To address this, we analyzed spending accumulated only in the first 6 months after treatment.

### 2.4 Statistical methods

The characteristics of ESCC patients were stratified by terciles, centers, and TNM stage. The analysis involved using the ANOVA test for continuous variables and the rank sum test for categorical variables.

We explored the temporal trend of spending by estimating the average spending per patient in each month following the onset first hospitalization, as shown in the following formula:


$$\begin{array}{l} \text{Average spending in a given month since first hospitalization}=\\ \frac{\text{Total spending in a given month}}{\text{number of alive patients in this month}} \end{array}$$

The given month was rounded to the next upward integer (for example, 7.8 months would be rounded up to 8 months).

OS was the primary outcome in our analysis, defined as the time interval from initial admission to either last contact or death. Median follow-up time and median survival time were estimated using the reverse Kaplan-Meier method and the Kaplan-Meier method, respectively. Kaplan-Meier survival curves were used to illustrate prognosis, and differences between curves were assessed with the log-rank test. Multivariable Cox proportional hazards models were used to calculate hazard ratios (HRs) and 95% confidence intervals (CIs) for comparing OS between patients in tercile 1 (lowest spending) and those in tercile 2, as well as between tercile 1 and tercile 3. Stratified analyses were performed by TNM stage and study center. To evaluate the independent association between spending and OS, we incorporated seven factors in the survival analysis: age, sex, occupation, stage, therapeutic approaches, number of hospital admissions, and length of hospital days. These factors were chosen from an initial set of 13 potential variables, which also considered comorbidities, postoperative complications, type of operation, health insurance status, family history of EC, and study center. All variables were initially evaluated using univariate Cox proportional hazards regression analysis. Those with a *P*-value<0.05 were further analyzed with multivariable Cox regression, employing backward selection (*P* < 0.05) to identify potential risk factors for OS. The therapeutic approaches included surgery alone, surgery combined with chemotherapy, surgery with radiotherapy, surgery with chemoradiotherapy, chemotherapy alone, radiotherapy alone, and chemoradiotherapy, with no fixed sequence within treatment plans.

All statistical analyses were performed using STATA version 15.1 (STATA, College Station, Texas, USA). All tests were two-sided and had a significance level of 0.05.

## 3 Results

### 3.1 Characteristics of ESCC patients

As shown in **Table 1**, most of the 11,037 ESCC patients were older than 60 years (70.6%), male (66.9%), and predominantly engaged in farming (60.1%). The majority were classified as stage III-IV (36.9%), underwent surgery alone (36.6%), and had a single hospital admission (46.7%). From tercile 1 to tercile 3, higher spending was associated with more advanced TNM stages (stage III: 25.0% vs. 37.6% vs. 48.1%; stage IV: 6.9% vs. 6.9% vs. 9.6%), more comprehensive treatment, and variations in age, sex, occupation, therapeutic approaches, and number of hospital admissions.

**Table 1:** Characteristics^a^ and mean spending^b^ of 11,037 selected patients with ESCC by spending tercile, China, 2009-2018.

Characteristics	Total		Tercile 1		Tercile 2		Tercile 3	
	n (%)	Mean USD	n (%)	Mean USD	n (%)	Mean USD	n (%)	Mean USD
Age at diagnosis (years)								
Median (quartile)	64(58,69)	19,461	65(59,71)	10,012	64(59,70)	16,511	62(57,67)	31,859
Age range at diagnosis (years)								
<60	3,249(29.4)	21,451	933(25.4)	9,863	1,001(27.2)	16,667	1,315(35.7)	33,314
≥60	7,788(70.6)	18,631	2,746(74.6)	10,062	2,678(72.8)	16,453	2,364(64.3)	31,050
Sex								
Male	7,385(66.9)	20,169	2,254(61.3)	9,943	2,485(67.6)	16,587	2,646(71.9)	32,244
Female	3,652(33.1)	18,029	1,425(38.7)	10,121	1,194(32.4)	16,353	1,033(28.1)	30,875
Occupation								
Farmer	6,639(60.1)	18,524	2,458(66.8)	10,125	2,162(58.8)	16,365	2,019(54.9)	31,062
Non-Farmer	2,747(24.9)	22,668	562(15.3)	9,983	980(26.6)	16,901	1,205(32.8)	33,275
Unemployment	1,651(15.0)	17,891	659(17.9)	9,615	537(14.6)	16,388	455(12.4)	31,649
Stage								
0-I	1,339(12.1)	14,450	762(20.7)	10,478	432(11.7)	15,853	145(3.9)	31,144
II	3,140(28.5)	18,391	1,156(31.4)	10,700	1,105(30.0)	16,312	879(23.9)	31,120
III	4,073(36.9)	21,900	921(25.0)	10,390	1,382(37.6)	16,748	1,770(48.1)	31,911
IV	862(7.8)	21,252	255(6.9)	8,180	253(6.9)	16,773	354(9.6)	33,869
Unknown	1,623(14.7)	18,591	585(15.9)	8,247	507(13.8)	16,729	531(14.4)	31,765
Therapeutic approaches								
Surgery	4,044(36.6)	15,253	2,035(55.3)	10,624	1,450(39.4)	15,969	559(15.2)	30,250
Surgery and chemotherapy	2,013(18.2)	23,584	269(7.3)	11,391	719(19.5)	17,144	1,025(27.9)	31,301
Surgery and radiotherapy	502(4.6)	24,987	24(0.7)	12,015	162(4.4)	17,214	316(8.6)	29,958
Surgery and chemoradiotherapy	622(5.6)	32,730	0(0.0)	NA^c^	44(1.2)	18,364	578(15.7)	33,824
Radiotherapy	903(8.2)	13,490	541(14.7)	9,578	271(7.4)	15,640	91(2.5)	30,342
Chemotherapy	689(6.2)	14,038	413(11.2)	6,203	122(3.3)	16,951	154(4.2)	32,744
Chemoradiotherapy	2,264(20.6)	22,471	397(10.8)	10,371	911(24.8)	16,861	956(26.0)	32,842
Hospital admissions								
1	5,153(46.7)	12,844	3,022(82.1)	10,071	1,911(51.9)	15,788	220(6.0)	25,356
2	1,972(17.9)	19,350	387(10.5)	9,802	833(22.6)	16,813	752(20.4)	27,074
3	1,114(10.1)	21,145	165(4.5)	9,256	447(12.2)	17,493	502(13.7)	28,304
4	676(6.1)	24,494	60(1.6)	9,667	204(5.5)	17,776	412(11.2)	29,980
≥5	2,122(19.2)	33,144	45(1.2)	11,049	284(7.7)	18,037	1,793(48.7)	36,092
Hospital days								
Mean (SD)	56.37(0.4)	19,461	29.82(0.2)	10,012	45.72(0.3)	16,511	93.57(0.7)	31,859

a The P values for patient characteristics, including age at diagnosis, age range, sex, occupation, stage, therapeutic approaches, hospital admissions, and hospital days, were all less than 0.001 across terciles 1, 2, and 3. The ANOVA test was used for categorical variables, and the rank sum test was applied to continuous variables.

b All costs were reported in US Dollars based on the 2023 value.

c No subjects in tercile 1 underwent surgery and chemoradiotherapy.

ESCC, esophageal squamous cell carcinoma; USD, United States dollars; NA, not applicable.

Patients at the Southern Center were younger (median age: 61 vs. 65), had a higher percentage of males (75.8% vs. 61.0%), and a lower proportion of farmers (16.9% vs. 89.1%) and cases treated with surgery only (24.4% vs. 44.8%). Additionally, they had a higher proportion of advanced-stage cases (stage III: 52.7% vs. 26.3%; stage IV: 9.7% vs. 6.5%) and longer hospital stays (64.3 days vs. 51.0 days) compared to patients at the Northern Center (**Supplementary Table S2**).

### 3.2 Spending of ESCC patients

As shown in **Table 1**, the mean spending per ESCC patient was $19,461, which increased by 218% from tercile 1 to tercile 3 ($10,012, $16,511, $31,859). Higher costs were associated with more advanced TNM stages (stage 0-I: $14,450; stage II: $18,391; stage III: $21,900; stage IV: $21,252). Spending also varied by age, sex, occupation, therapeutic approaches, and number of hospital admissions. Except for tercile 1, which had lower costs for advanced TNM stages, tercile 2 and tercile 3 exhibited similar patterns. These trends were consistent across both the Southern and Northern Centers (**Supplementary Table S2**).

A higher degree of treatment complexity was associated with increased spending. As shown in **Supplementary Table S3**, the proportion of stage 0-II patients undergoing surgery alone sharply decreased from tercile 1 to tercile 3 (83.0% vs. 73.0% vs. 40.6%). Patients in higher spending terciles tended to receive more comprehensive treatments, such as additional adjuvant therapy. Similarly, **Supplementary Table S4** shows that a similar trend was observed among stage III-IV patients undergoing surgery alone, with proportions decreasing from tercile 1 to tercile 3 (35.99% vs. 21.70% vs. 7.90%).

**Figure 2:**
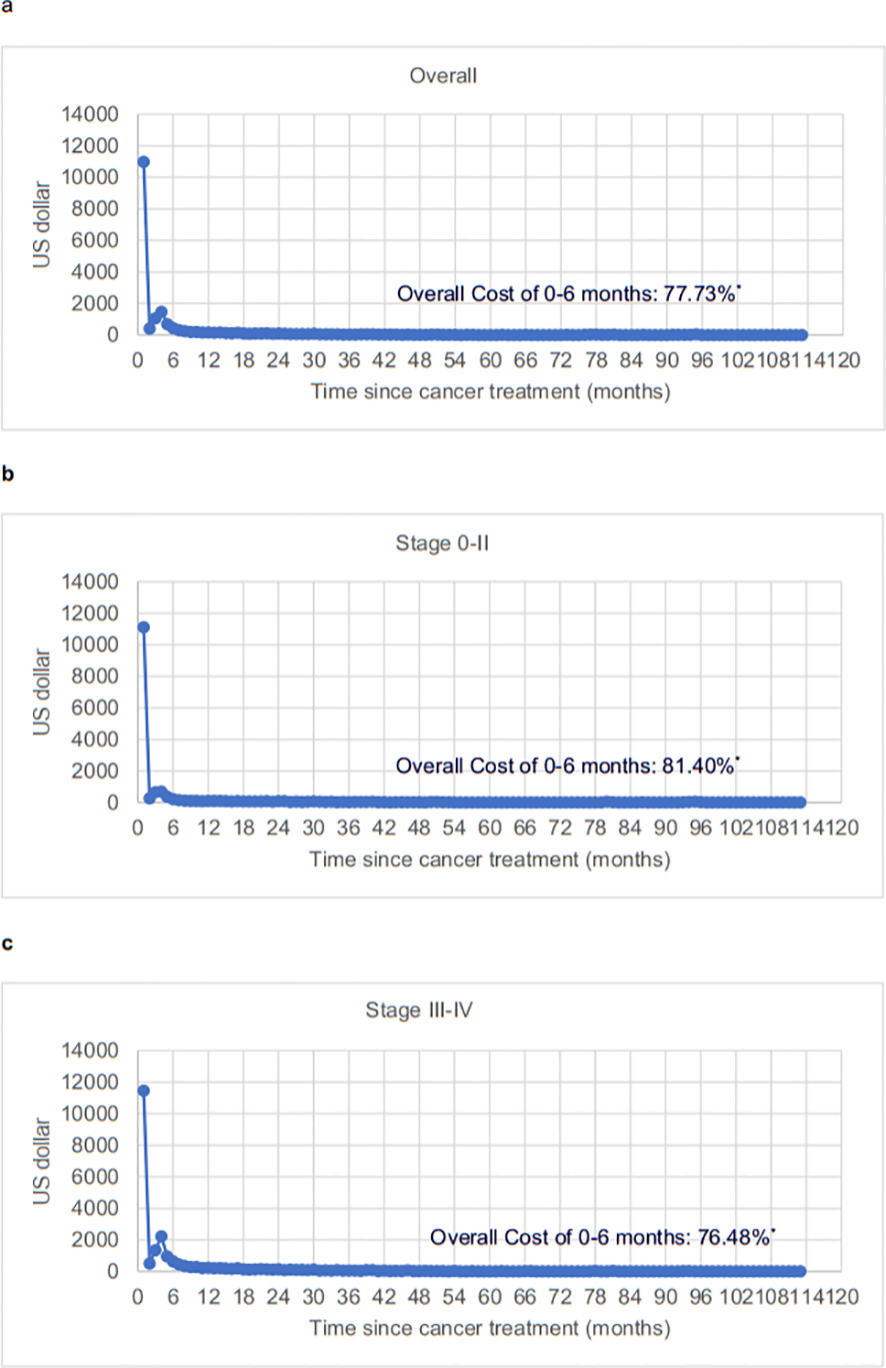
Time trends for monthly average hospital spending per ESCC patient since first hospitalization. [**(A)** Overall ESCC patients; **(B)** Stage 0-II ESCC patients; **(C)** Stage III-IV ESCC patients]. All costs were reported in US dollars based on the 2023 value. *The proportional of life-time costs within 6 months. ESCC, esophageal squamous cell carcinoma.

As shown in **Figure 1**, the costs associated with drugs, surgery, non-surgical treatments, inspections, and other expenses were 38%, 7%, 24%, 13%, and 18%, respectively. Drug costs constituted the largest portion for all patients (38%), the Southern Center (36%), and the Northern Center (39%). **Figure 2** illustrates that the time trends for monthly average hospital spending since the first hospitalization followed an “L-shaped” pattern, with the majority of lifetime hospital spending incurred within the first six months (overall: 77.73%; stage 0-II: 81.40%; stage III-IV: 76.48%). This trend remained consistent across patients stratified by study center (**Supplementary Figure S2**).

As shown in **Supplementary Tables S3** and **S4**, mean hospital spending for ESCC patients, stratified by stage, varied significantly based on the type of therapy received. Surgery combined with adjuvant therapy was more costly than surgery alone. For patients with stage 0-II, the spending on surgery combined with chemoradiotherapy, surgery with radiotherapy, surgery with chemotherapy, and surgery alone was $32,225, $23,584, $21,521, and $14,917, respectively. In comparison, spending for patients with stage III-IV was $32,992, $25,504, $25,230, and $16,247, respectively. Additionally, chemoradiotherapy was more expensive than both radiotherapy and chemotherapy (stage 0-II: $20,684 vs. $13,304 and $19,425; stage III-IV: $23,061 vs. $13,751 and $15,066).

### 3.3 Association between hospital spending and OS of ESCC patients

**Figure 3:**
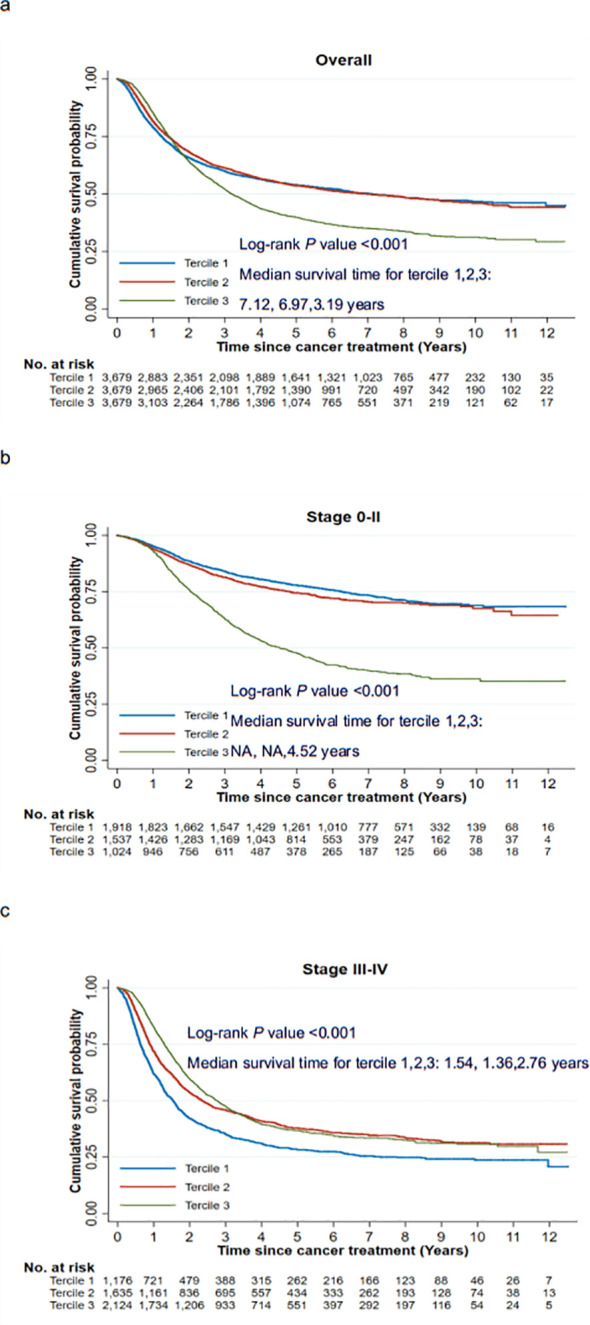
Kaplan-Meier survival curves for ESCC patients stratified by spending quartile. [**(A)** Kaplan-Meier survival curves of OS; **(B)** Kaplan-Meier survival curves for stage 0-II ESCC; **(C)** Kaplan-Meier survival curves for stage III-IV ESCC]. ESCC, esophageal squamous cell carcinoma; HR, hazard ratio; OS, overall survival.

After a maximum follow-up of 12.52 years, the median follow-up time, median survival time, and age-standardized (ASR) 5-year survival rate of ESCC patients was 6.79 years (95% CI: 6.68-6.85 years), 4.70 years (95% CI: 4.39-5.10 years), and 49.58% (95% CI: 47.90%-51.23%), respectively. As shown in **Figure 3**, Kaplan-Meier survival curves for ESCC patients showed tercile 3 (median survival time:3.19 years) was linked to worse OS compared with tercile 1 (median survival time:6.97 years) and tercile 2 (median survival time:7.12 years). This pattern was consistent for stage 0-II patients but reversed for those with stage III-IV. Similar patterns were observed in the Kaplan-Meier survival curves stratified by study center (**Supplementary Figure S3**).

**Figure 4:**
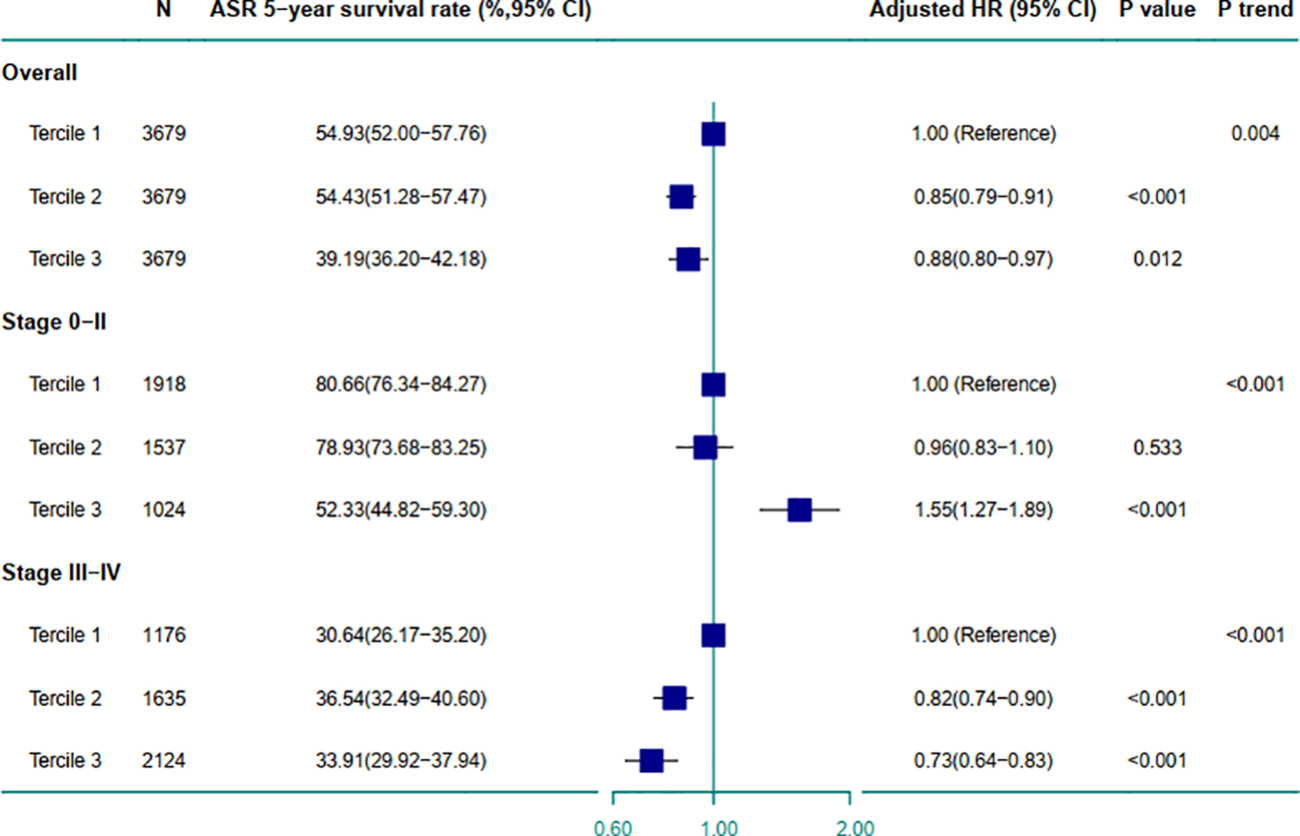
Comparison of ESCC patient survival between terciles stratified by TNM stage. The HRs and *P* values were calculated from multivariable Cox regression models adjusting for age, sex, occupation, therapy approaches, number of clinical visits, length of stay. The *P* trend value was derived by treating the ordinal variable as a continuous variable. Error bars represent 95% CI for HR estimates. ASR 5-year survival rate, age-standardized relative 5-year survival rate; ESCC, esophageal squamous cell carcinoma; HR, hazard ratio.

**Figure 4** illustrates that multivariable Cox regression models demonstrated higher spending was associated with better OS (adjusted HR_tercile 2 vs 1_ = 0.85, 95% CI: 0.79-0.91; adjusted HR_tercile 3 vs 1_ = 0.88, 95% CI: 0.80-0.97). These findings were consistent for patients with stage III-IV (adjusted HR_tercile 2 vs 1_ = 0.82, 95% CI: 0.74-0.90; adjusted HR_tercile 3 vs 1_ = 0.73, 95% CI: 0.64-0.83), but reversed for those with stage 0-II, where higher spending was associated with worse OS (adjusted HR_tercile 3 vs 1_ = 1.55, 95% CI: 1.27-1.89). Similar patterns were observed in the multivariable Cox regression models stratified by study center (**Supplementary Figure S4**).

### 3.4 Sensitivity analysis

The findings from the indirect perspective were consistent with those from the direct perspective (**Supplementary Materials**, **Supplementary Table S5**, **Supplementary Figures S5**-**S8**). Similarly, the analysis of spending accumulated only within the first 6 months also yielded consistent results, supporting the robustness of our primary findings (**Supplementary Materials**, **Supplementary Table S6**, **Supplementary Figures S9**, **10**).

## 4 Discussion

Improving survival and reducing costs in cancer treatment has become an increasingly recognized priority (9, 10). However, the value of cancer treatment varied by cancer types, tumor stages and types of therapy (13–16, 22), with higher spending often linked to more comprehensive treatments but not necessarily to improvements in OS (23). This trend is particularly evident in advanced-stage colorectal, breast, and head and neck cancers (13–16). In contrast, higher spending was associated with better OS in lung cancer treatment, which was attributed to targeted services provided according to guidelines (17). To our knowledge, only one previous study has explored the value of neoadjuvant treatment in ESCC (11). To further investigate, we conducted this prospective study involving 11,037 ESCC patients, with a median follow-up time of 6.79 years, to explore the association between hospital spending and OS for ESCC patients who underwent different treatments. Our findings indicated that higher spending was associated with better OS in the overall population of ESCC patients, particularly those with advanced-stage ESCC, while higher spending was linked to worse OS in early-stage ESCC patients.

In our study, higher spending was associated with more comprehensive treatment both in early-stage and advanced-stage patients, e.g., patients with higher spending were more likely to receive additional adjuvant therapy. This difference of clinical benefits between early-stage and advanced-stage ESCC patients was caused by the unbalance between the clinical benefits and toxicities of comprehensive treatment. For early-stage ESCC patients, additional adjuvant therapy only increased treatment toxicity without providing clinical benefits (24). In contrast, for advanced-stage ESCC patients, adjuvant therapy could help reduce locoregional recurrence, prevent distant metastasis, and improve OS (25). Similar findings were observed in our previous cohort study of ESCC patients, which demonstrated that clinical benefits and treatment toxicities varied by stage (18). These results provide evidence supporting the importance of standardized treatment for ESCC and the improvement of health insurance reimbursement policies.

This study was conducted at two Grade-A tertiary cancer hospitals, offering comprehensive cancer treatment services within their catchment regions. However, the unbalance between clinical benefits and treatment toxicities for the treatment of early-stage patients still happened. They may potentially administer overly intensive, non-targeted treatments to early-stage cancer patients. Unfortunately, our previous study also found that some clinicians from high-risk ESCC regions lacked basic knowledge of early-stage treatment guidelines (26). Furthermore, some early-stage patients, anxious about their cancer diagnosis, may disregard clinical guidance and opt for more extensive treatments if they can afford them (9). Therefore, there is an urgent need for continuous medical education on early-stage cancer treatment guidelines, particularly for clinicians.

In contrast, the reasons of advanced-stage ESCC patients who underwent insufficient treatment were caused by low socioeconomic status (26) and high treatment costs (16), particularly when they are from resource-limited areas. Given that the risk of ESCC is strongly associated with low socioeconomic status (27, 28) and that most high-risk regions for ESCC are rural areas (1, 2), the tendency to refuse treatment may be more common in the patients from these regions. In our previous work, we observed farmers with advanced-stage ESCC were forced to sell possessions and into debt until they could no longer afford the expensive comprehensive treatment. Although, the insurance coverage reached over 95% of the Chinese population in 2013, and has been sustained since (29). Out-of-pocket expenses for EC treatment remain high, and current insurance schemes are insufficient for EC patients to access necessary treatment, particularly for advanced-stage ESCC patients (6).

To enhance the value of treatment both for these early-stage and advanced-stage ESCC patients, here are three recommendations for policymaking. First, oncology training for clinicians should be strengthened in alignment with established guidelines. This training should emphasize early diagnosis, the avoidance of overtreatment for early-stage patients, and the provision of optimal care for those with advanced-stage disease, including current nutritional support (30) and future consideration of techniques like robotic-assisted esophagectomy to improve outcomes (31). Second, health insurance reimbursement should be strictly aligned with standardized ESCC treatment guidelines for both early-stage and advanced-stage patients. Where feasible, reimbursement levels for advanced-stage patients in high-incidence regions could be increased to support comprehensive treatment, especially high drug costs which constituted the largest portion for all patients, and to ensure coverage of subsequent therapies after discharge by the national health system or personal insurance plans, which is crucial for guaranteeing the best possible patient survival outcomes. Third, adequate education on the clinical benefits and treatment toxicities of ESCC should be provided for cancer patients to prevent nonstandard treatments.

Unexpectedly, we found that the lifetime costs for ESCC patients in China followed an “L-shaped” pattern in both this study and our previous randomized controlled trial in rural areas (32). In contrast, end-of-life costs for EC patients in the US exhibited a “U-shaped” pattern (33). This disparity may be attributed to the lower socioeconomic status (26) and older age of most ESCC patients, which often limits their families’ ability to afford continuous anti-cancer treatment, leading to the forgoing of life-sustaining care due to financial burdens in China (34). Furthermore, Chinese cancer patients from rural areas prefer end-of-life care and dying at home rather than in a hospital setting (35, 36).

A limitation of this study is the unavailability of outpatient cost data, we likely underestimated the total treatment costs. Hopefully, since outpatient expenses typically account for less than 10% of overall cancer treatment costs (37), their exclusion is unlikely to significantly affect the findings of our study. Additionally, complication data were only available for patients after surgery, comprising 64.49% of the cohort. To address potential confounding from complications and comorbidities, we provided comparative data across spending groups (**Supplementary Tables S7** and **S8**). Our analysis indicated that the association between higher spending and increased complication rates was observed solely among early-stage surgical patients. This finding underscores the need for future research to determine the direction and mechanism of this relationship.

## 5 Conclusion

In summary, our investigation found that higher spending was associated with worse OS in early-stage ESCC patients, while it was linked to better OS in those with advanced-stage ESCC. These findings support the need for tailored clinical decision-making and stage-based improvements in health insurance, ensuring more conservative therapeutic approaches for early-stage ESCC patients and sufficient treatments for advanced-stage patients.

## Data Availability

The raw data supporting the conclusions of this article will be made available by the authors, without undue reservation.
