# Supplementary material for: Could higher hospital spending improve survival in patients with esophageal squamous cell carcinoma? A multicenter retrospective cohort study
Source: Front Oncol. 2026 Jan 22;15:1668017. doi: 10.3389/fonc.2025.1668017 (PMC12873576; doi:10.3389/fonc.2025.1668017)
Supplement: Supplementary file 1 [file Supplementaryfile1.docx]

# Supplementary materials

## Data collection and processing

The electronic clinical records were exported from the Hospital Information System (HIS), capturing variables of interest such as demographics (age, sex, etc.), tumor characteristics (TNM stage [determined according to the 7th edition of the American Joint Committee on Cancer Staging Manual]^1^, etc.), treatment-related data (surgical or internal medicine, therapeutic approaches, etc.). The clinical database consisted of both structured and unstructured data. Two trained researchers manually extracted the unstructured data following specified standardized procedures, using Epidata software (version 3.1, EpiData Association, Odense, Denmark). Random sampling of unstructured data revealed a discrepancy of less than 5% between the two data extractors, and final results were confirmed by checking original medical records.

A follow-up database system was utilized at the Southern Center to automate the extraction of patient lists from HIS. Follow-up lists that require immediate action can be generated. Full-time personnel performed annual telephone follow-ups, inputting data such as follow-up dates, patient physical condition, survival information, etc., into the system. Moreover, the system automatically retrieved information on patients’ routine reexamination status after initial treatment from the HIS, thereby enhancing the follow-up data.

## Sensitivity analysis

Cost calculation were conducted from indirect perspective in sensitivity analysis, accounting for the sum of hospital spending and time costs. The time cost was evaluated by the Human Capital Approach based on Overall Length of Stay (OLS) and Annual Net Income (ANI) per capita for residents at the Southern Center and Northern Center (TableS1). Spending for a caregiver was taken into consideration and the time cost for each hospitalization was estimated from Eq. (1) as follows

$$\text{Time cost=OLS×}\frac{\text{ANI}}{\text{365}}\text{×2}$$

All costs were reported in Chinese Yuan (CNY) based on the 2023 value, which were inflated using the year-specific personal health care consumer price index (CPI) of Southern Center (Shantou city) and Northern Center (Anyang city), respectively (TableS1). Then, they were converted from Chinese Yuan to US Dollars using purchasing power parity exchange rates of 2023 ($1 = ¥3.64)^2^.

Patients with ESCC firstly admitted to Southern Center (2009.08-2018.12)

(N=5,232)

Total population: ESCC patients treated for the first time (N=11,037)

Patients with ESCC firstly admitted to Northern Center (2012.01-2017.12)

(N=9,127)

Excluded

·Not anti-cancer treatment (N=543)

·<6 months follow-up (N=259)

Excluded

·Not anti-cancer treatment (N=561)

·Unavailable cost data (N=14)

·<6 months follow-up (N=1,945)

## **FigureS1. Flow diagram of patients with ESCC in the Southern Center and Northern Center**.

Abbreviations: ESCC, esophageal squamous cell carcinoma.

a b

Overall Cost of 0-6 months: 78.34%^*^

Overall Cost of 0-6 months: 83.09%^*^

c d

Overall Cost of 0-6 months: 82.55%^*^

Overall Cost of 0-6 months: 74.48%^*^

## **FigureS2. Time trends for monthly average hospital spending per ESCC patient since first hospitalization.** [(A) Stage 0-II ESCC patients in the Southern Center; (B) Stage III-IV ESCC patients in the Southern Center; (C) Stage 0-II ESCC patients in the Northern Center; (D) Stage III-IV ESCC patients in the Northern Center].

Notes: All costs were reported in US dollars based on the 2023 value, which were inflated using the year-specific personal health care consumer price index (CPI) of Southern Center (Shantou city) and Northern Center (Anyang city), respectively. We explored the temporal trend of spending by estimating the average spending per patient in each month after the first hospitalization began, as in the following formula:

$$\text{Average spending in a given month since first hospitalization=}$$

$$\frac{\text{Total spending in a given month}}{\text{number of alive patients in this month}}$$

The given month was rounded to the next upward integer (for example, 7.8 months would be rounded up to 8 months).

*The proportional of life-time cost within 6 months.

Abbreviations: ESCC, esophageal squamous cell carcinoma.

a b

c
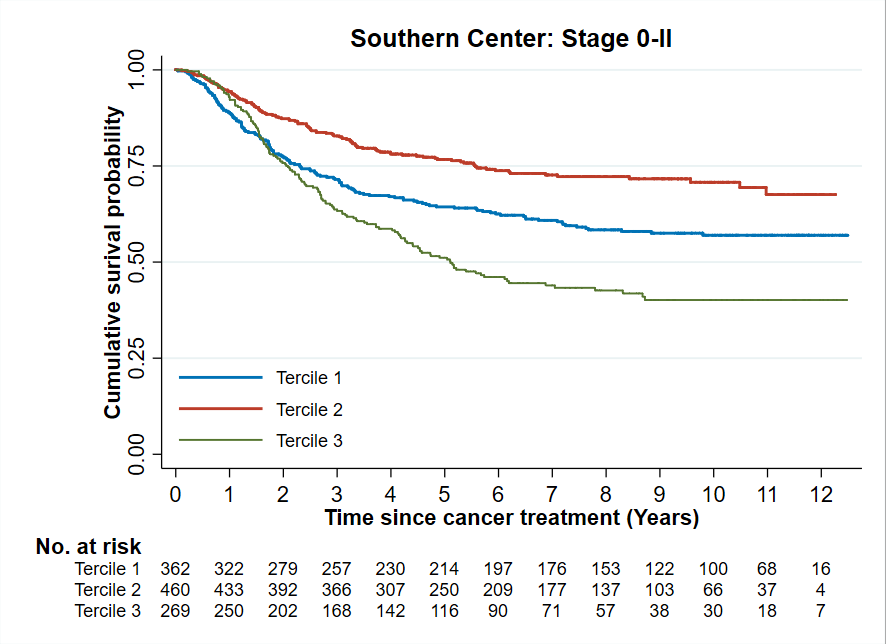
 d
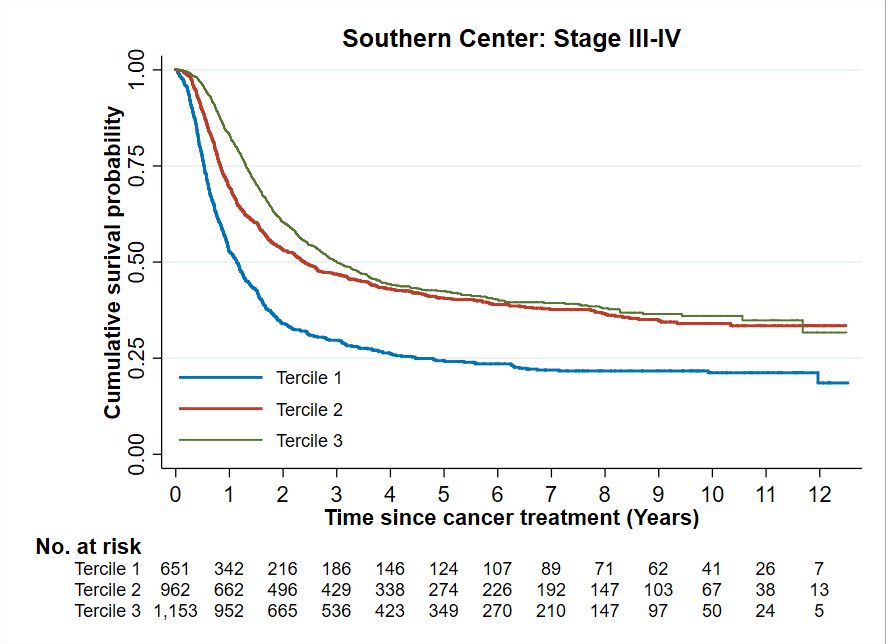


Log-rank *P* value <0.001

Median survival time for tercile 1,2,3:

1.14, 2.40,3.00 years

Log-rank *P* value <0.001

Median survival time for tercile 1,2,3:

NA, NA,5.11 years


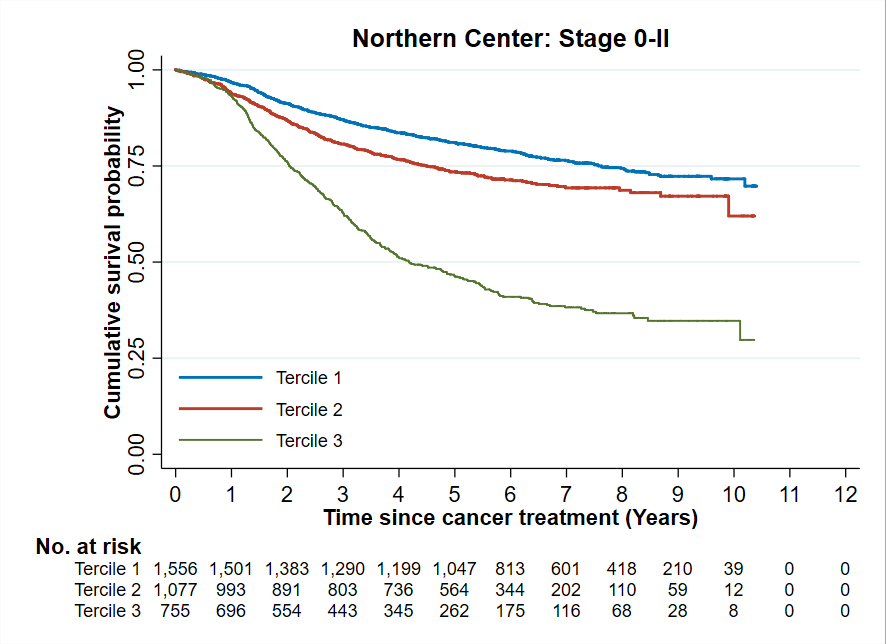

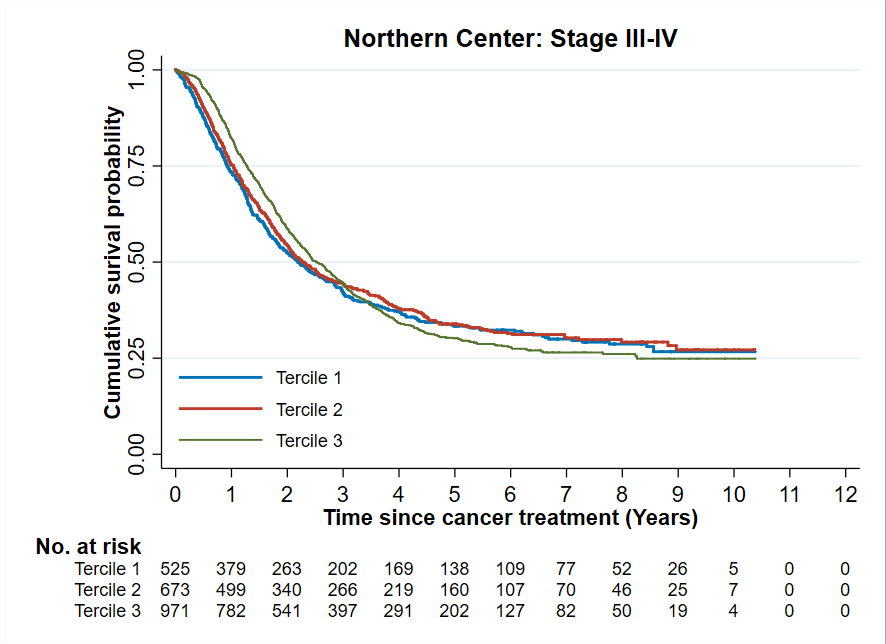


Log-rank *P* value <0.001

Median survival time for tercile 1,2,3:

2.19, 2.27,2.52 years

Log-rank *P* value <0.001

Median survival time for tercile 1,2,3:

NA, NA,4.18 years

## **FigureS3.** Kaplan-Meier survival curves for ESCC patients stratified by **spending quartile.** [(A) Kaplan-Meier survival curves of OS for stage 0-II ESCC in the Southern Center; (B) Kaplan-Meier survival curves of OS for stage III-IV ESCC in the Southern Center; (C) Kaplan-Meier survival curves of OS for stage 0-II ESCC in the Northern Center; (D) Kaplan-Meier survival curves of OS for stage III-IV ESCC in the Northern Center].

Notes: Tercile 1 indicated the lowest spending quartile, while tercile 3 was the highest spending quartile. The mean hospital spending of tercile 1 to tercile 3 are $10,012, $16,511, $31,859, respectively. The difference of survival among the terciles was compared using the log-rank test. Median survival time was estimated using the Kaplan-Meier method.

Abbreviations: OS, overall survival; ESCC, esophageal squamous cell carcinoma; HR: hazard ratio.


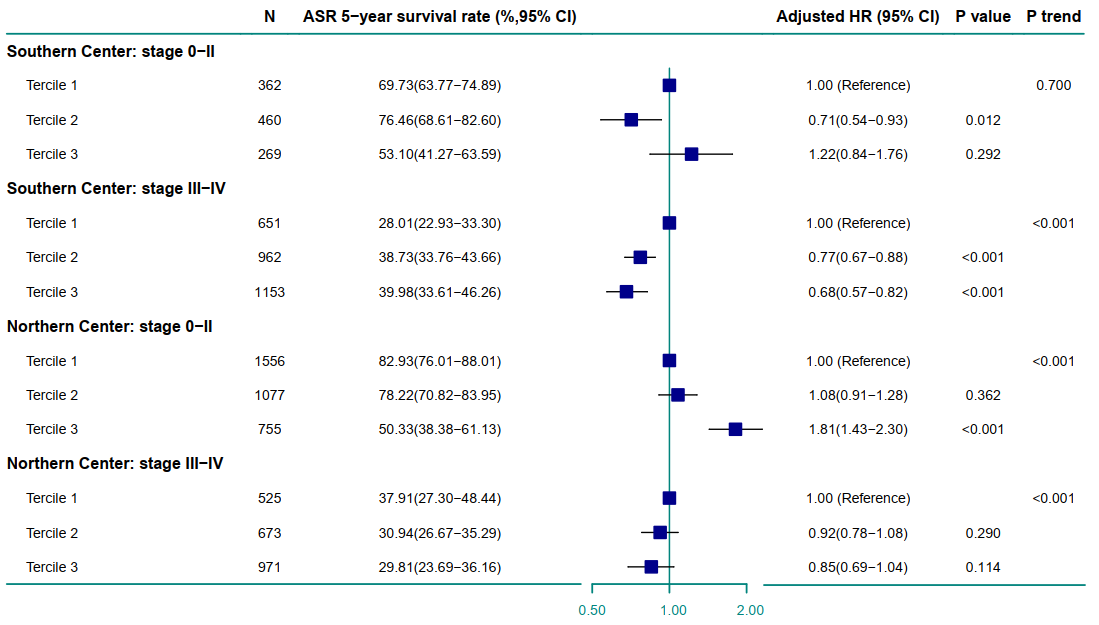


## FigureS4. Comparison of ESCC patient survival between Southern Center and Northern Center stratified by TNM stage and terciles.

Notes: HRs are shown for Tercile 1 through 3 in comparison with quartile 1 (reference). The HRs and *P* values were calculated from multivariable Cox regression models adjusting for age, sex, occupation, therapy approaches, number of clinical visits, length of stay. The P trend value was derived by treating the ordinal variable as a continuous variable. Error bars represent 95% CI for HR estimates. Tercile 1 indicated the lowest spending quartile, while tercile 3 was the highest spending quartile. The mean hospital spending of tercile 1 to tercile 3 are $10,012, $16,511, $31,859, respectively.

Abbreviations: ASR 5-year survival rate: age-standardized relative 5-year survival rate; ESCC, esophageal squamous cell carcinoma; HR: hazard ratio.

a

b

c

## FigureS5. The proportional breakdown of hospital spending for ESCC patients from indirect perspective. [(A) Overall; (B) Southern Center; (C) Northern Center].

Notes: Patients diagnosed with ESCC who were first admitted to hospital between August 1, 2009 and December 31, 2018 at the Southern Center (Cancer Hospital of Shantou University Medical College, Shantou City, Guangdong Province, China), and between January 1, 2012 and December 31, 2017 at the Northern Center (Anyang Cancer Hospital, Anyang City, Henan Province, China. Non-surgical treatment includes radiation, Chinese traditional medicine and oxygen therapy. Others include nursing, bed, laboratory test and diagnosis. All costs were reported in Chinese Yuan (CNY) based on the 2023 value, which were inflated using the year-specific personal health care consumer price index (CPI) of Southern Center (Shantou city) and Northern Center (Anyang city), respectively (TableS1). Then, they were converted from Chinese Yuan to US Dollars using purchasing power parity exchange rates of 2023 ($1 = ¥3.64)^2^.

Abbreviations: ESCC, esophageal squamous cell carcinoma.

a

b

c

## **FigureS6. Time trends for monthly average hospital spending per ESCC patient since first hospitalization.** [(A) Overall ESCC patients; (B) Stage 0-II ESCC patients; (C) Stage III-IV ESCC patients].

Notes: All costs were reported in US dollars based on the 2023 value, which were inflated using the year-specific personal health care consumer price index (CPI) of Southern Center (Shantou city) and Northern Center (Anyang city), respectively. We explored the temporal trend of spending by estimating the average spending per patient in each month after the first hospitalization began, as in the following formula:

$$\text{Average spending in a given month since first hospitalization=}$$

$$\frac{\text{Total spending in a given month}}{\text{number of alive patients in this month}}$$

The given month was rounded to the next upward integer (for example, 7.8 months would be rounded up to 8 months).

*The proportional of life-time costs within 6 months.

Abbreviations: ESCC, esophageal squamous cell carcinoma.

a


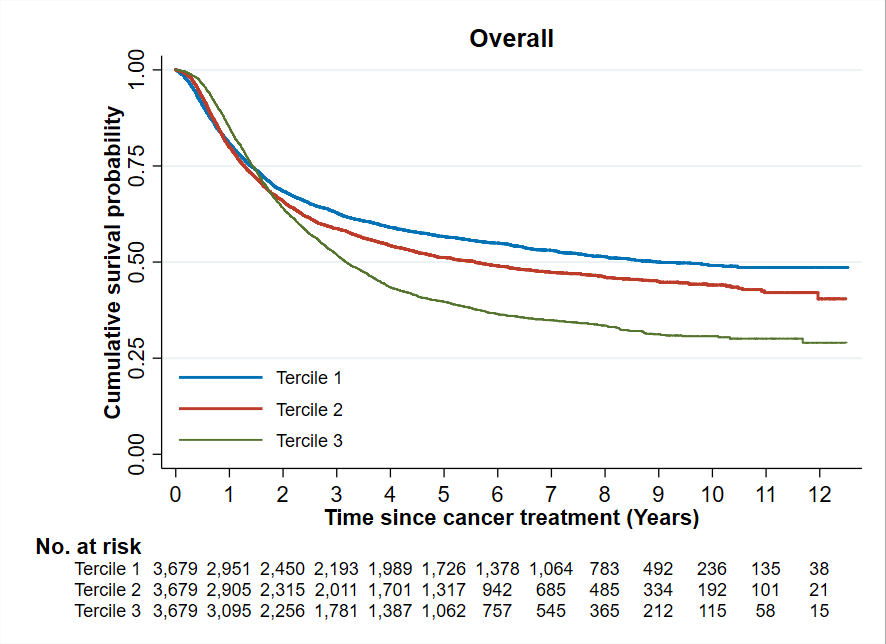


Log-rank *P* value <0.001

Median survival time for tercile 1,2,3: 8.96, 5.58,3.17 years

b


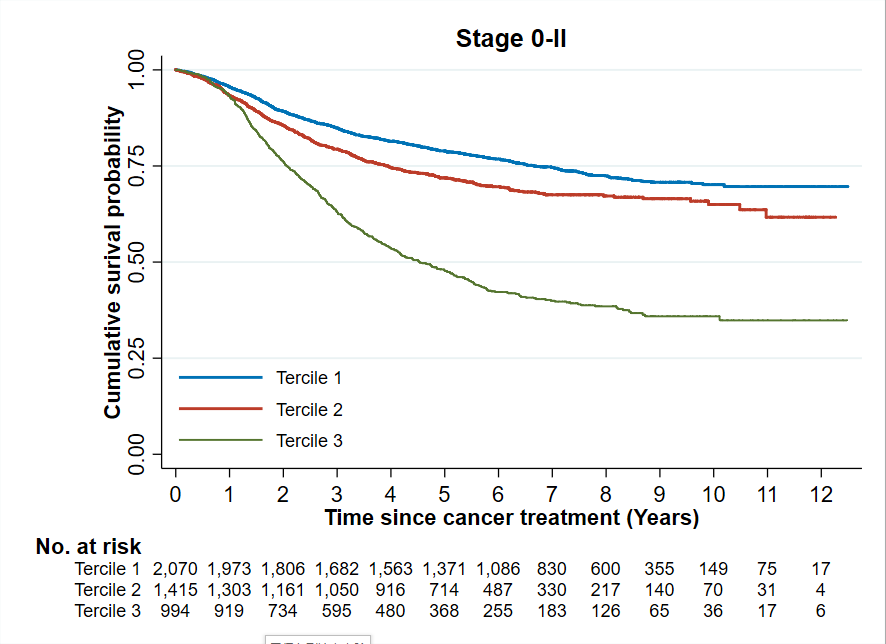


Log-rank *P* value <0.001

Median survival time for tercile 1,2,3: NA, NA,4.53 years

c


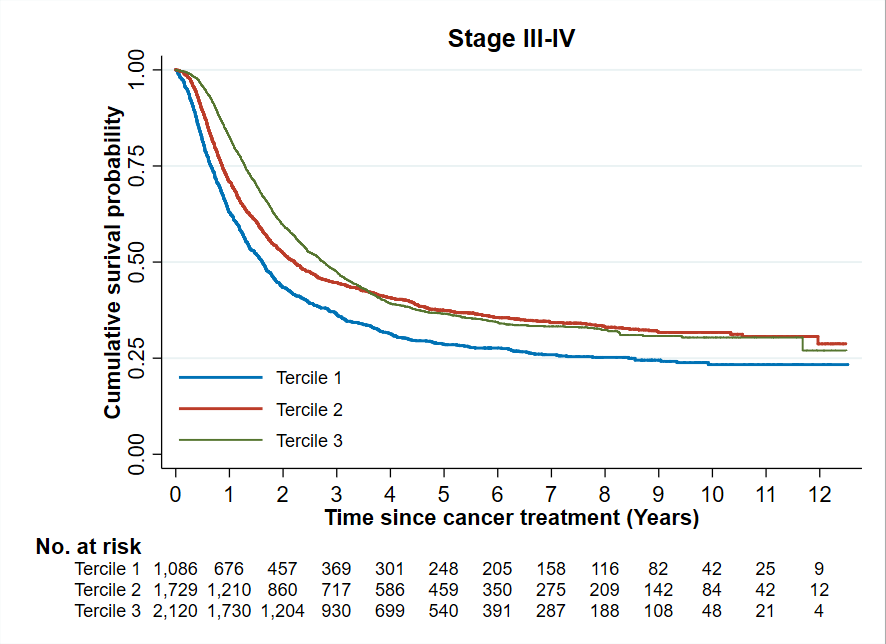


Log-rank *P* value <0.001

Median survival time for tercile 1,2,3: 1.61, 2.22,2.75 years

## **FigureS7.** Kaplan-Meier survival curves for ESCC patients stratified by **spending quartile from indirect perspective.** [(A) Kaplan-Meier survival curves of OS; (B) Kaplan-Meier survival curves for stage 0-II ESCC; (C) Kaplan-Meier survival curves for stage III-IV ESCC].

Notes: Tercile 1 indicated the lowest spending quartile, while tercile 3 was the highest spending quartile. From the direct perspective, the mean hospital spending of overall patients for tercile 1 ,2 and 3 were $10,012, $16,511, $31,859, respectively. The difference of survival among the terciles was compared using the log-rank test. Median survival time was estimated using the Kaplan-Meier method

Abbreviations: OS, overall survival; ESCC, esophageal squamous cell carcinoma; HR: hazard ratio.


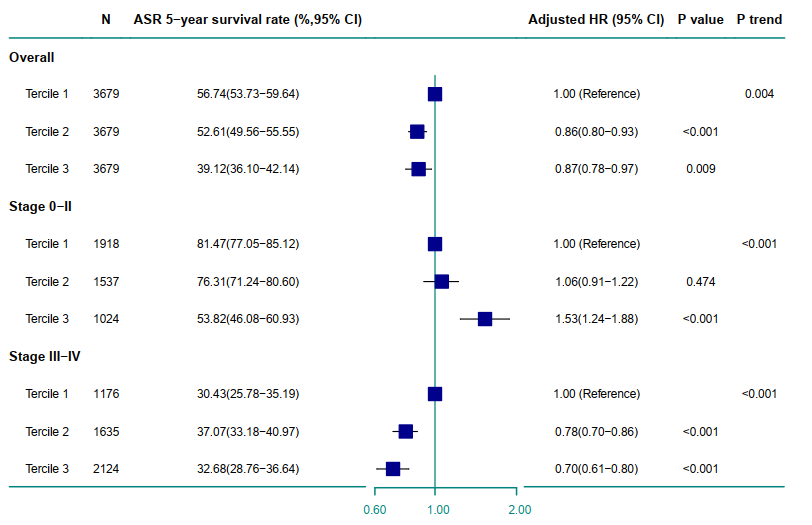


## **FigureS8.** Comparison of ESCC patient survival between terciles stratified by TNM stage from indirect perspective.

Notes: HRs are shown for Tercile 1 through 3 in comparison with quartile 1 (reference). The HRs and P values were calculated from multivariable Cox regression models adjusting for age, sex, occupation, therapy approaches, number of clinical visits, length of stay. The P trend value was derived by treating the ordinal variable as a continuous variable. Error bars represent 95% CI for HR estimates. Tercile 1 indicated the lowest spending quartile, while tercile 3 was the highest spending quartile. The mean hospital spending of tercile 1 to tercile 3 are $10,012, $16,511, $31,859, respectively.

Abbreviations: ASR 5-year survival rate: age-standardized relative 5-year survival rate; ESCC, esophageal squamous cell carcinoma; HR: hazard ratio.

a


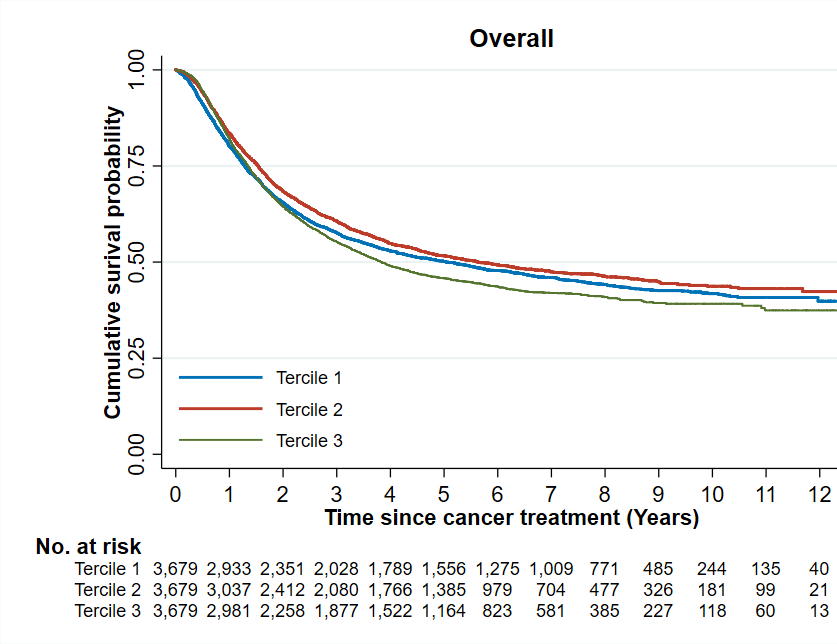


Log-rank *P* value <0.001

Median survival time for tercile 1,2,3: 5.04,5.64,3.83years

b


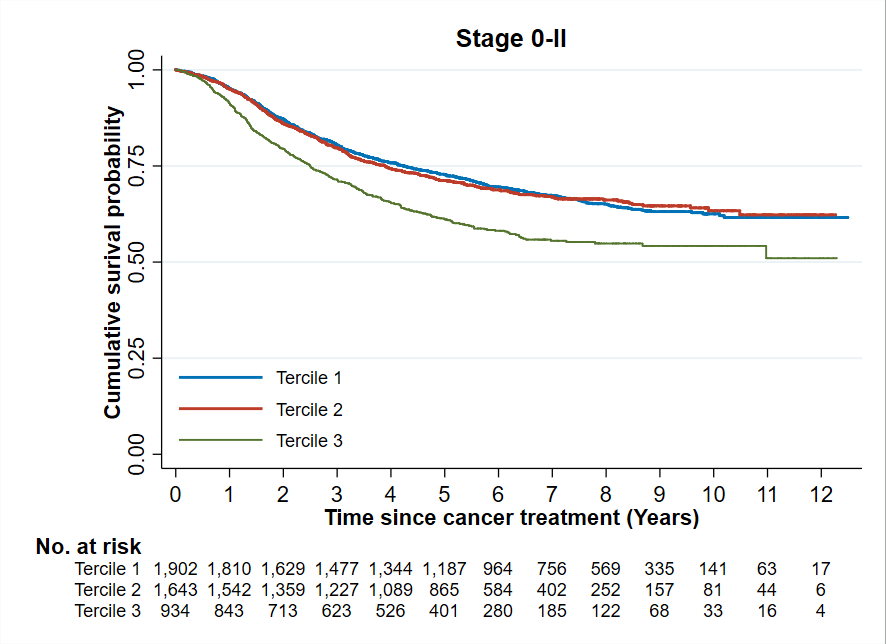


Log-rank *P* value <0.001

Median survival time for tercile 1,2,3: NA,NA,NA years

c


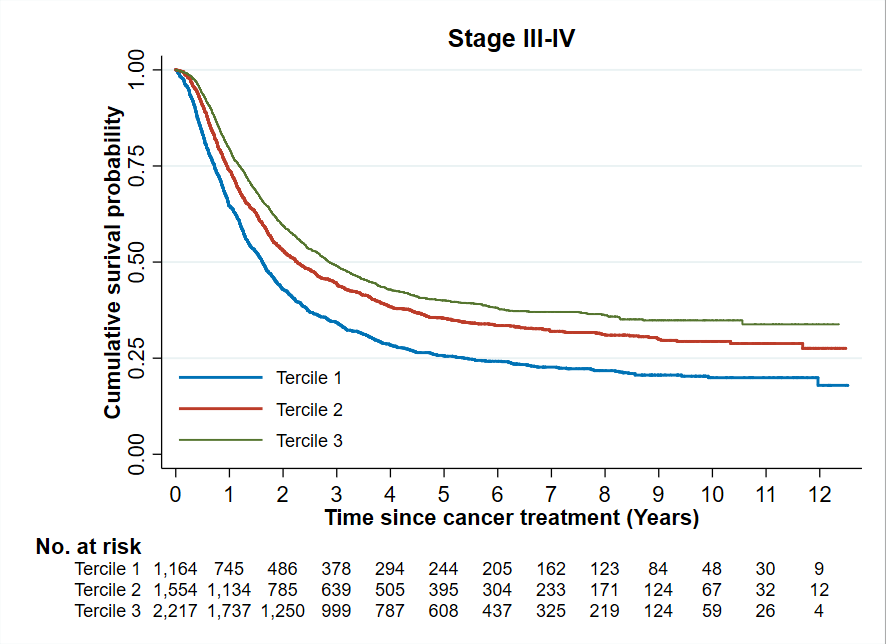


Log-rank *P* value <0.001

Median survival time for tercile 1,2,3: 1.61, 2.27,2.86 years

## **FigureS9.** Kaplan-Meier survival curves for ESCC patients stratified by **spending quartile.** [(A) Kaplan-Meier survival curves of OS; (B) Kaplan-Meier survival curves for stage 0-II ESCC; (C) Kaplan-Meier survival curves for stage III-IV ESCC].

Notes: The spending was accumulated only in the first 6 months. Tercile 1 indicated the lowest spending quartile, while tercile 3 was the highest spending quartile. From the direct perspective, the mean hospital spending of overall patients for tercile 1 ,2 and 3 were $9,530, $14,999, $24,595, respectively. The difference of survival among the terciles was compared using the log-rank test. Median survival time was estimated using the Kaplan-Meier method

Abbreviations: OS, overall survival; ESCC, esophageal squamous cell carcinoma; HR: hazard ratio.


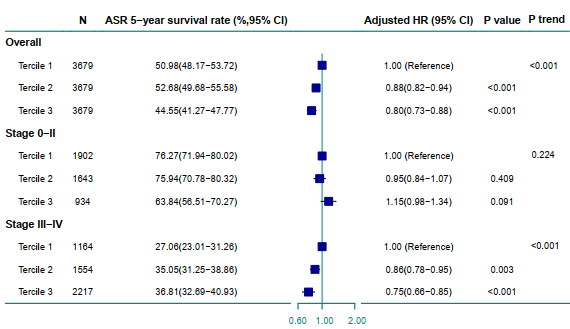


## **FigureS10.** Comparison of ESCC patient survival between terciles stratified by TNM stage.

Notes: The spending was accumulated only in the first 6 months. HRs are shown for Tercile 1 through 3 in comparison with quartile 1 (reference). The HRs and P values were calculated from multivariable Cox regression models adjusting for age, sex, occupation, therapy approaches, number of clinical visits, length of stay. The P trend value was derived by treating the ordinal variable as a continuous variable. Error bars represent 95% CI for HR estimates. Tercile 1 indicated the lowest spending quartile, while tercile 3 was the highest spending quartile. The mean hospital spending of tercile 1 to tercile 3 are $10,012, $16,511, $31,859, respectively.

Abbreviations: ASR 5-year survival rate: age-standardized relative 5-year survival rate; ESCC, esophageal squamous cell carcinoma; HR: hazard ratio.

## TableS1. Basic socio-economic statistics adopted in cost calculations.

| Year | Southern Center^a^ | | Northern Center^a^ | |
| --- | --- | --- | --- | --- |
|  | Annual Net Income per capita of residents in Anyang (CNY) ^b,c^ | Health care Consumer Price Index in Shantou city^c^ | Annual Net Income per capita of residents in Shantou (CNY) ^b,c^ | Health care Consumer Price Index in Anyang city^c^ |
| 2009 | 25,482 | 102.4 | NA | NA |
| 2010 | 27,918 | 107.1 | NA | NA |
| 2011 | 32,844 | 105.2 | NA | NA |
| 2012 | 37,328 | 100.8 | 33,925 | 101.9 |
| 2013 | 42,286 | 103 | 34,613 | 102.2 |
| 2014 | 45,455 | 101.6 | 39,820 | 100.7 |
| 2015 | 52,299 | 103.7 | 42,272 | 101.7 |
| 2016 | 55,129 | 102.9 | 46,412 | 101.4 |
| 2017 | 60,901 | 106.7 | 51,098 | 104.1 |
| 2018 | 68,100 | 104.7 | 61,079 | 107.1 |
| 2019 | 75,468 | 105.8 | 62,727 | 102 |
| 2020 | 82,833 | 98.8 | 70,239 | 104.6 |
| 2021 | 85,920 | 100.3 | 71,955 | 102.8 |
| 2022 | 91,667 | 99.9 | 72,315 | 100.5 |
| 2023 | 95,982 | 101.6 | 73,023 | 101.1 |

^a^ Patients were recruited between August 1, 2009 and December 31, 2018 at the Southern Center (Cancer Hospital of Shantou University Medical College, Shantou City, Guangdong Province, China), and between January 1, 2012 and December 31, 2017 at the Northern Center (Anyang Cancer Hospital, Anyang City, Henan Province, China).

^b^ All costs were reported in Chinese Yuan (CNY).

^C^ The data of Annual Net Income per capita of residents and Health care Consumer Price Index were exported from the Bureau of Statistics in Anyang City and Shantou City, respectively.

## TableS2. Characteristics^a^ and mean spending^b^ of the 11,037 selected patients with esophageal squamous cell carcinoma, China, 2009-2018.

|  | **Total** | | **Southern Center** | | **Northern Center** | |
| --- | --- | --- | --- | --- | --- | --- |
|  | **n (%)** | **USD** | **n (%)** | **USD** | **n (%)** | **USD** |
| **Age at diagnosis (years)** |  |  |  |  |  |  |
| Median (quartile) | 64(58,69) | 19,461 | 61(56,68) | 20,216 | 65(60,70) | 18,955 |
| **Age range at diagnosis (years)** |  |  |  |  |  |  |
| <60 | 3249(29.4) | 21,451 | 1806(40.8) | 22,447 | 1443(21.8) | 20,204 |
| ≥60 | 7788(70.6) | 18,631 | 2624(59.2) | 18,680 | 5164(78.2) | 18,606 |
| **Sex** |  |  |  |  |  |  |
| Male | 7385(66.9) | 20,169 | 3358(75.8) | 20,922 | 4027(61.0) | 19,541 |
| Female | 3652(33.1) | 18,029 | 1072(24.2) | 18,003 | 2580(39.1) | 18,039 |
| **Occupation** |  |  |  |  |  |  |
| Farmer | 6639(60.1) | 18,524 | 750(16.9) | 19,694 | 5889(89.1) | 18,375 |
| Non-Farmer | 2747(24.9) | 22,668 | 2043(46.1) | 22,289 | 704(10.7) | 23,770 |
| Unemployment | 1651(15.0) | 17,891 | 1637(37.0) | 17,868 | 14(0.2) | 20,560 |
| **Stage** |  |  |  |  |  |  |
| 0-I | 1339(12.1) | 14,450 | 251(5.7) | 15,332 | 1088(16.5) | 14,247 |
| II | 3140(28.5) | 18,391 | 840(19.0) | 18,429 | 2300(34.8) | 18,378 |
| III | 4073(36.9) | 21,900 | 2336(52.7) | 21,801 | 1737(26.3) | 22,033 |
| IV | 862(7.8) | 21,252 | 430(9.7) | 21,252 | 432(6.5) | 21,252 |
| Unknown | 1623(14.7) | 18,591 | 573(12.9) | 17,734 | 1050(15.9) | 19,059 |
| **Therapeutic approaches**^c^ |  |  |  |  |  |  |
| Surgery | 4044(36.6) | 15,253 | 1082(24.4) | 16,653 | 2962(44.8) | 14,742 |
| Surgery and chemotherapy | 2013(18.2) | 23,584 | 285(6.4) | 26,999 | 1728(26.2) | 23,021 |
| Surgery and radiotherapy | 502(4.6) | 24,987 | 380(8.6) | 24,461 | 122(1.9) | 26,625 |
| Surgery and chemoradiotherapy | 622(5.6) | 32,730 | 379(8.6) | 32,611 | 243(3.6) | 32,916 |
| Radiotherapy | 903(8.2) | 13,490 | 639(14.4) | 12,387 | 264(4.0) | 16,159 |
| Chemotherapy | 689(6.2) | 14,038 | 163(3.7) | 13,256 | 526(8.0) | 14,281 |
| Chemoradiotherapy | 2264(20.6) | 22,471 | 1502(33.9) | 21,379 | 762(11.5) | 24,624 |
| **Hospital admissions** |  |  |  |  |  |  |
| 1 | 5153(46.7) | 12,844 | 2043(26.1) | 13,387 | 3110(47.1) | 12,487 |
| 2 | 1972(17.9) | 19,350 | 989(22.3) | 20,934 | 983(14.9) | 17,757 |
| 3 | 1114(10.1) | 21,145 | 463(10.5) | 22,841 | 651(9.9) | 19,938 |
| 4 | 676(6.1) | 24,494 | 234(5.3) | 26,534 | 442(6.6) | 23,414 |
| ≥5 | 2122(19.2) | 33,144 | 701(15.8) | 35,261 | 1421(21.5) | 32,100 |
| **Hospital days** |  |  |  |  |  |  |
| Mean (SD) | 56.37(38.9) | 19,461 | 64.31(38.4) | 20,216 | 51.04(38.3) | 18,955 |

^a^ The P values for patient characteristics, including age at diagnosis, age range, sex, occupation, stage, therapeutic approaches, hospital admissions, and hospital days, were all less than 0.001 between Southern Center and Northern Center. The ANOVA test was used for categorical variables, and the rank sum test was applied to continuous variables.

^b^ All costs were reported in Chinese Yuan (CNY) based on the 2023 value, which were inflated using the year-specific personal health care consumer price index (CPI) of Southern Center (Shantou city) and Northern Center (Anyang city), respectively (TableS1). Then, they were converted from Chinese Yuan to US Dollars using purchasing power parity exchange rates of 2023 ($1 = ¥3.64)^2^.

^c^ “Surgery and chemotherapy” means patients undergoing both surgery and chemotherapy without a predetermined sequence, so is “surgery and radiotherapy”, “surgery and chemoradiotherapy”.

## TableS3. Characteristics^a^ and mean spending^b^ of the 4,479 stage 0-II patients by spending tercile for esophageal squamous cell carcinoma, China, 2009-2018.

|  | **Total** | | **Tercile 1** ^a^ | | **Tercile 2** | | **Tercile 3** | |  |  |  |
| --- | --- | --- | --- | --- | --- | --- | --- | --- | --- | --- | --- |
|  | **n (%)** | **USD** | **n (%)** | **USD** | **n (%)** | **USD** | **n (%)** | **USD** |  |  |  |
| **Age at diagnosis (years)** | | |  |  |  |  |  |  |  |  |  |
| Median (quartile) | 64(59,69) | 17213 | 64(59,69) | 9998 | 65(60,69) | 14384 | 63(58,68) | 27257 |  |  |  |
| **Age range at diagnosis (years)** | | |  |  |  |  |  |  |  |  | |
| <60 | 1,224(27.3) | 18151 | 407(27.3) | 9762 | 351(23.5) | 14448 | 466(31.2) | 28266 |  |  |  |
| ≥60 | 3,255(72.7) | 16861 | 1,086(72.7) | 10086 | 1,142(76.5) | 14365 | 1,027(68.8) | 26800 |  |  |  |
| **Sex** |  |  |  |  |  |  |  |  |  |  |  |
| Male | 2,749(61.4) | 17687 | 840(56.3) | 9979 | 894(59.9) | 14454 | 1,015(68.0) | 26913 |  |  |  |
| Female | 1,730(38.6) | 16460 | 653(43.7) | 10022 | 599(40.1) | 14280 | 478(32.0) | 27988 |  |  |  |
| **Occupation** | |  |  |  |  |  |  |  |  |  |  |
| Farmer | 3,209(71.7) | 16556 | 1,177(78.8) | 10065 | 1,107(74.2) | 14248 | 925(62.0) | 27575 |  |  |  |
| Non-Farmer | 893(19.9) | 19650 | 182(12.2) | 9849 | 270(18.9) | 14856 | 441(29.5) | 26630 |  |  |  |
| Unemployment | 377(8.4) | 17038 | 134(9.0) | 9608 | 116(7.8) | 14582 | 127(8.5) | 27120 |  |  |  |
| **Therapeutic approaches^a^** | | |  |  |  |  |  |  |  |  | |
| Surgery | 2,935(65.5) | 14917 | 1,239(83.0) | 10027 | 1,090(73.0) | 14296 | 606(40.6) | 26035 |  |  |  |
| Surgery and chemotherapy | 914(20.4) | 21521 | 125(8.4) | 10589 | 267(17.9) | 14744 | 522(35.0) | 27605 |  |  |  |
| Surgery and radiotherapy | 127(2.8) | 23584 | 3(0.2) | 10588 | 30(2.0) | 14923 | 94(6.3) | 26764 |  |  |  |
| Surgery and chemoradiotherapy | 114(2.6) | 32225 | 0(0.0) | NA^c^ | 1(0.1) | 14762 | 113(7.6) | 32379 |  |  |  |
| Radiotherapy | 137(3.1) | 13304 | 79(5.3) | 9254 | 36(2.4) | 13942 | 22(1.5) | 26803 |  |  |  |
| Chemotherapy | 47(1.1) | 19425 | 14(0.9) | 5895 | 9(0.6) | 14337 | 24(1.6) | 29225 |  |  |  |
| Chemoradiotherapy | 205(4.6) | 20684 | 33(2.2) | 10139 | 60(4.0) | 14384 | 112(7.5) | 27166 |  |  |  |
| **Hospital admissions** | |  |  |  |  |  |  |  |  |  |  |
| 1 | 2,819(62.9) | 12806 | 1,381(92.5) | 9992 | 1,151(77.1) | 14219 | 287(19.2) | 20678 |  |  |  |
| 2 | 641(14.3) | 18793 | 90(6.0) | 10156 | 226(15.1) | 14748 | 325(21.8) | 23997 |  |  |  |
| 3 | 285(6.4) | 22193 | 13(0.9) | 10051 | 67(4.5) | 15136 | 205(13.7) | 25269 |  |  |  |
| 4 | 175(3.9) | 25729 | 6(0.4) | 10791 | 24(1.6) | 15238 | 145(9.7) | 28084 |  |  |  |
| ≥5 | 559(12.5) | 32425 | 3(0.2) | 6304 | 25(1.7) | 15878 | 531(35.6) | 33352 |  |  |  |
| **Hospital days** | |  |  |  |  |  |  |  |  | |  |
| Mean (SD) | 43.79(0.5) | 17213 | 24.50(0.3) | 9998 | 31.69(0.3) | 14384 | 75.19(1.1) | 27257 |  |  |  |

^a^ The P values for patient characteristics, including age at diagnosis, age range, sex, occupation, stage, therapeutic approaches, hospital admissions, and hospital days, were all less than 0.001 across terciles 1, 2, and 3. The ANOVA test was used for categorical variables, and the rank sum test was applied to continuous variables.

^b^ All costs were reported in Chinese Yuan (CNY) based on the 2023 value, which were inflated using the year-specific personal health care consumer price index (CPI) of Southern Center (Shantou city) and Northern Center (Anyang city), respectively (TableS1). Then, they were converted from Chinese Yuan to US Dollars using purchasing power parity exchange rates of 2023 ($1 = ¥3.64)^2^.

^c^ Tercile 1 indicated the lowest spending quartile, while tercile 3 was the highest spending quartile.

^d^ “Surgery and chemotherapy” means patients undergoing both surgery and chemotherapy without a predetermined sequence, so is “surgery and radiotherapy”, “surgery and chemoradiotherapy”.

^e^ No subjects in tercile 1 underwent surgery and chemoradiotherapy.

## TableS4. Characteristics^a^ and mean spending^b^ of the 4,935 stage III-IV patients by spending tercile for esophageal squamous cell carcinoma, China, 2009-2018.

|  | **Total** | | **Tercile 1** ^c^ | | **Tercile 2** | | **Tercile 3** | |
| --- | --- | --- | --- | --- | --- | --- | --- | --- |
|  | **n (%)** | **USD** | **n (%)** | **USD** | **n (%)** | **USD** | **n (%)** | **USD** |
| **Age at diagnosis (years)** | | |  |  |  |  |  |  |
| Median (quartile) | 63(57,68) | 21787 | 65(59,71) | 11146 | 63(58,68) | 19105 | 61(56,66) | 35109 |
| **Age range at diagnosis (years)** | | |  |  |  |  |  |  |
| <60 | 1,648(33.4) | 24108 | 444(27.0) | 11197 | 531(32.3) | 19219 | 673(40.9) | 36483 |
| ≥60 | 3,287(66.6) | 20623 | 1,201(73.0) | 11127 | 1,114(67.7) | 19051 | 972(59.1) | 34157 |
| **Sex** |  |  |  |  |  |  |  |  |
| Male | 3,565(72.2) | 22480 | 1,122(68.2) | 11143 | 1,179(71.7) | 19098 | 1,264(76.8) | 35698 |
| Female | 1,370(27.8) | 19983 | 523(31.8) | 11152 | 466(28.3) | 19124 | 381(23.2) | 33155 |
| **Occupation** | |  |  |  |  |  |  |  |
| Farmer | 2,470(50.1) | 21045 | 863(52.5) | 11174 | 835(50.8) | 19178 | 772(46.9) | 34098 |
| Non-Farmer | 1,594(32.3) | 24659 | 372(22.6) | 11253 | 538(32.7) | 19072 | 684(41.6) | 36345 |
| Unemployment | 871(17.7) | 18634 | 410(24.9) | 10990 | 272(16.5) | 18949 | 189(11.5) | 34760 |
| **Therapeutic approaches^d^** | | |  |  |  |  |  |  |
| Surgery | 1,079(21.9) | 16247 | 592(36.0) | 11598 | 357(21.7) | 18106 | 130(7.9) | 32317 |
| Surgery and chemotherapy | 1,030(20.9) | 25230 | 143(8.7) | 12782 | 432(26.3) | 19668 | 455(27.7) | 34423 |
| Surgery and radiotherapy | 368(7.5) | 25504 | 43(2.6) | 13575 | 141(8.6) | 19770 | 184(11.2) | 32686 |
| Surgery and chemoradiotherapy | 466(9.4) | 32992 | 2(0.1) | 14602 | 69(4.2) | 20766 | 395(24.0) | 35221 |
| Radiotherapy | 406(8.2) | 13751 | 304(18.5) | 10896 | 83(5.1) | 18136 | 19(1.2) | 40266 |
| Chemotherapy | 365(7.4) | 15066 | 222(13.5) | 7098 | 75(4.6) | 19041 | 68(4.1) | 36693 |
| Chemoradiotherapy | 1,221(24.7) | 23061 | 339(20.6) | 12213 | 488(29.7) | 19086 | 394(24.0) | 37318 |
| **Hospital admissions** | |  |  |  |  |  |  |  |
| 1 | 1,699(34.4) | 13425 | 1,179(71.7) | 11017 | 478(29.1) | 17890 | 42(2.6) | 30207 |
| 2 | 1,009(20.4) | 20505 | 261(15.9) | 11789 | 432(26.3) | 19129 | 315(19.2) | 29613 |
| 3 | 605(12.3) | 21527 | 117(7.1) | 10570 | 284(17.3) | 19186 | 204(12.4) | 31071 |
| 4 | 370(7.5) | 24720 | 41(2.5) | 10956 | 152(9.2) | 19730 | 177(10.8) | 32194 |
| ≥5 | 1,253(25.4) | 33414 | 47(2.9) | 12404 | 299(18.2) | 20618 | 907(55.1) | 38721 |
| **Hospital days** | |  |  |  |  |  |  |  |
| Mean (SD) | 64.34(0.6) | 21787 | 35.86(0.4) | 11146 | 55.12(0.5) | 19105 | 102.06(1.0) | 35109 |

^a^ The P values for patient characteristics, including age at diagnosis, age range, sex, occupation, stage, therapeutic approaches, hospital admissions, and hospital days, were all less than 0.001 across terciles 1, 2, and 3. The ANOVA test was used for categorical variables, and the rank sum test was applied to continuous variables.

^b^ All costs were reported in Chinese Yuan (CNY) based on the 2023 value, which were inflated using the year-specific personal health care consumer price index (CPI) of Southern Center (Shantou city) and Northern Center (Anyang city), respectively (TableS1). Then, they were converted from Chinese Yuan to US Dollars using purchasing power parity exchange rates of 2023 ($1 = ¥3.64)^2^.

^c^ Tercile 1 indicated the lowest spending quartile, while tercile 3 was the highest spending quartile.

^d^ “Surgery and chemotherapy” means patients undergoing both surgery and chemotherapy without a predetermined sequence, so is “surgery and radiotherapy”, “surgery and chemoradiotherapy”.

## TableS5. Mean spending^a^ of the 11,037 selected patients with ESCC by spending tercile from indirect perspective, China, 2009-2018.

|  | **Total** | **Tercile 1^b^** | **Tercile 2** | **Tercile 3** |
| --- | --- | --- | --- | --- |
| **Overall** | 24,177 | 12,211 | 20,377 | 39,944 |
| **Age range at diagnosis (years)** |  |  |  |  |
| <60 | 26,723 | 11,957 | 20,545 | 41,576 |
| ≥60 | 23,115 | 12,297 | 20,316 | 39,015 |
| **Sex** |  |  |  |  |
| Male | 25,090 | 12,156 | 20,448 | 40,388 |
| Female | 22,332 | 12,296 | 20,228 | 38,802 |
| **Occupation** |  |  |  |  |
| Farmer | 22,737 | 12,235 | 20,342 | 38,925 |
| Non-Farmer | 28,589 | 12,282 | 20,617 | 41,857 |
| Unemployment | 22,627 | 12,045 | 20,086 | 39,247 |
| **Stage** |  |  |  |  |
| 0-I | 17,238 | 12,548 | 19,378 | 38,925 |
| II | 22,339 | 12,876 | 20,023 | 38,997 |
| III | 27,365 | 12,679 | 20,691 | 40,104 |
| IV | 27,314 | 9,939 | 21,025 | 42,740 |
| Unknown | 23,794 | 10,296 | 20,580 | 39,332 |
| **Therapeutic approaches^c^** |  |  |  |  |
| Surgery | 18,103 | 12,713 | 19,492 | 38,193 |
| Surgery and chemotherapy | 28,713 | 13,571 | 21,109 | 39,412 |
| Surgery and radiotherapy | 31,714 | 14,220 | 21,133 | 37,438 |
| Surgery and chemoradiotherapy | 40,865 | NA^d^ | 23,302 | 42,170 |
| Radiotherapy | 18,031 | 12,318 | 19,318 | 37,273 |
| Chemotherapy | 17,785 | 7,919 | 21,252 | 41,411 |
| Chemoradiotherapy | 29,135 | 12,748 | 21,002 | 40,916 |
| **Hospital admissions** |  |  |  |  |
| 1 | 15,614 | 12,246 | 19,428 | 31,764 |
| 2 | 24,228 | 11,959 | 20,720 | 33,502 |
| 3 | 26,601 | 11,769 | 21,507 | 35,333 |
| 4 | 30,732 | 12,206 | 22,113 | 37,816 |
| ≥5 | 41,564 | 13,704 | 22,445 | 45,537 |

^a^ All costs were reported in Chinese Yuan (CNY) based on the 2023 value, which were inflated using the year-specific personal health care consumer price index (CPI) of Southern Center (Shantou city) and Northern Center (Anyang city), respectively (TableS1). Then, they were converted from Chinese Yuan to US Dollars using purchasing power parity exchange rates of 2023 ($1 = ¥3.64).

^b^ Tercile 1 indicated the lowest spending quartile, while tercile 3 was the highest spending quartile.

^c^ “Surgery and chemotherapy” means patients undergoing both surgery and chemotherapy without a predetermined sequence, so is “surgery and radiotherapy”, “surgery and chemoradiotherapy”.

^d^ No subjects in tercile 1 underwent surgery and chemoradiotherapy.

Abbreviations: ESCC, esophageal squamous cell carcinoma; NA, not applicable.

## TableS6. Characteristics^a^ and mean spending^b^ of the 11,037 selected patients with ESCC, China, 2009-2018.

|  | **Total** | | **Tercile 1** | | **Tercile 2** | | **Tercile 3** | |
| --- | --- | --- | --- | --- | --- | --- | --- | --- |
|  | **n (%)** | **USD** | **n (%)** | **USD** | **n (%)** | **USD** | **n (%)** | **USD** |
| **Age at diagnosis (years)** | | |  |  |  |  |  |  |
| Median (quartile) | 64(58,69) | 16,375 | 64(59,70) | 9,530 | 64(59,70) | 14,999 | 62(57,67) | 24,595 |
| **Age range at diagnosis (years)** | | |  |  |  |  |  |  |
| <60 | 3,249(29.4) | 17,473 | 971(26.4) | 9,436 | 994(27.0) | 15,148 | 1,284(34.9) | 25,352 |
| ≥60 | 7,788(70.6) | 15,916 | 2,708(73.6) | 9,564 | 2,685(73.0) | 14,945 | 2,395(65.1) | 24,189 |
| **Sex** |  |  |  |  |  |  |  |  |
| Male | 7,385(66.9) | 16,914 | 2,284(62.1) | 9,450 | 2,416(65.7) | 15,066 | 2,685(73.0) | 24,926 |
| Female | 3,652(33.1) | 15,284 | 1,395(37.9) | 9,660 | 1,263(34.3) | 14,871 | 994(27.0) | 23,700 |
| **Occupation** | |  |  |  |  |  |  |  |
| Farmer | 6,639(60.1) | 15,719 | 2,423(65.9) | 9,640 | 2,226(60.5) | 14,856 | 1,990(54.1) | 24,088 |
| Non-Farmer | 2,747(24.9) | 18,798 | 588(16.0) | 9,506 | 891(24.2) | 15,455 | 1,268(34.5) | 25,457 |
| Unemployment | 1,651(15.0) | 14,978 | 668(18.2) | 9,151 | 562(15.3) | 14,847 | 421(11.4) | 24,399 |
| **Stage** |  |  |  |  |  |  |  |  |
| 0-I | 1,339(12.1) | 12,988 | 712(19.4) | 10,034 | 488(13.3) | 14,612 | 139(3.8) | 22,416 |
| II | 3,140(28.5) | 15,379 | 1,190(32.4) | 10,279 | 1,155(31.4) | 14,907 | 795(21.6) | 23,698 |
| III | 4,073(36.9) | 18,568 | 897(24.4) | 9,832 | 1,316(35.8) | 15,122 | 1,860(50.6) | 25,219 |
| IV | 862(7.8) | 17,110 | 267(7.3) | 7,707 | 238(6.5) | 15,307 | 357(9.7) | 25,343 |
| Unknown | 1,623(14.7) | 15,202 | 613(16.7) | 7,842 | 482(13.1) | 15,126 | 528(14.4) | 23,815 |
| **Therapeutic approaches** ^c^ | | |  |  |  |  |  |  |
| Surgery | 4,044(36.6) | 13,168 | 2,039(55.4) | 10,214 | 1,611(43.8) | 14,763 | 394(10.7) | 21,932 |
| Surgery and chemotherapy | 2,013(18.2) | 19,459 | 245(6.7) | 10,713 | 644(17.5) | 15,397 | 1,124(30.6) | 23,693 |
| Surgery and radiotherapy | 502(4.6) | 21,157 | 26(0.7) | 10,711 | 132(3.6) | 15,245 | 344(9.4) | 24,215 |
| Surgery and chemoradiotherapy | 622(5.6) | 28,820 | 5(0.1) | 10,446 | 29(0.8) | 16,037 | 588(16.0) | 29,606 |
| Radiotherapy | 903(8.2) | 11,909 | 522(14.2) | 9,100 | 319(8.7) | 14,425 | 62(1.7) | 22,623 |
| Chemotherapy | 689(6.2) | 10,905 | 445(12.1) | 6,031 | 109(3.0) | 15,007 | 135(3.7) | 23,659 |
| Chemoradiotherapy | 2,264(20.6) | 18,327 | 397(10.8） | 9,685 | 835(22.7) | 15,292 | 1,032(28.1) | 24,107 |
| **Hospital admissions** | |  |  |  |  |  |  |  |
| 1 | 5,153(46.7) | 12,844 | 2,598(70.6) | 9,601 | 2,057(55.9) | 14,740 | 498(13.5) | 21,931 |
| 2 | 1,972(17.9) | 17,220 | 509(13.8) | 9,214 | 700(19.0) | 15,139 | 763(20.7) | 24,471 |
| 3 | 1,114(10.1) | 18,187 | 237(6.4) | 9,184 | 350(9.5) | 15,492 | 527(14.3) | 24,027 |
| 4 | 676(6.1) | 19,616 | 119(3.2) | 9,491 | 174(4.7) | 15,471 | 383(10.4) | 24,646 |
| ≥5 | 2,122(19.2) | 22,179 | 216(5.9) | 9,826 | 398(10.8) | 15,455 | 1,508(41.0) | 25,723 |
| **Hospital days** | |  |  |  |  |  |  |  |
| Mean (SD) | 56.37(0.4) | 16,375 | 29.00(0.2) | 9,530 | 40.86(0.3) | 14,999 | 68.09(0.4) | 24,595 |

^a^ The P values for patient characteristics, including age at diagnosis, age range, sex, occupation, stage, therapeutic approaches, hospital admissions, and hospital days, were all less than 0.001 between Southern Center and Northern Center. The ANOVA test was used for categorical variables, and the rank sum test was applied to continuous variables.

^b^ The spending was accumulated only in the first 6 months. All costs were reported in Chinese Yuan (CNY) based on the 2023 value, which were inflated using the year-specific personal health care consumer price index (CPI) of Southern Center (Shantou city) and Northern Center (Anyang city), respectively (TableS1). Then, they were converted from Chinese Yuan to US Dollars using purchasing power parity exchange rates of 2023 ($1 = ¥3.64)^2^.

^c^ “Surgery and chemotherapy” means patients undergoing both surgery and chemotherapy without a predetermined sequence, so is “surgery and radiotherapy”, “surgery and chemoradiotherapy”.

## TableS7. Comorbidities of the 11,037 selected patients with ESCC by spending tercile, China, 2009-2018.

|  | Tercile 1 | Tercile 2 | Tercile 3 | *χ²* | *P* value^a^ |
| --- | --- | --- | --- | --- | --- |
| Overall patients |  |  |  |  |  |
| with comorbidities | 1,248(33.92%) | 1,224(33.27%) | 1,192(32.40%) | 1.93 | 0.380 |
| without comorbidities | 2,431(66.08%) | 2,455(66.73%) | 2,487(67.60%) |  |  |
| Stage 0-II |  |  |  |  |  |
| with comorbidities | 715(37.28%) | 578(37.61%) | 396(38.67%) | 0.56 | 0.755 |
| without comorbidities | 1,203(62.72%) | 959(62.39%) | 628(61.33%) |  |  |
| Stage III-IV |  |  |  |  |  |
| with comorbidities | 373(31.72%) | 483(29.54%) | 639(30.08%) | 1.61 | 0.447 |
| without comorbidities | 803(68.28%) | 1,152(70.46%) | 1,485(69.92%) |  |  |

^a^ The ANOVA test was used to calculate *p* value. Abbreviations: ESCC, esophageal squamous cell carcinoma

## TableS8. Postoperative complication rates of the 7,181 selected patients with ESCC by spending tercile, China, 2009-2018.

|  | Tercile 1 | Tercile 2 | Tercile 3 | *χ²* | *P* value^a^ |
| --- | --- | --- | --- | --- | --- |
| Overall patients |  |  |  |  |  |
| with complication | 447(31.79%) | 490(34.85%) | 469(33.36%) | 2.55 | 0.279 |
| without complication | 1,881(80.80%) | 1,885(79.37%) | 2,009(81.07%) |  |  |
| Stage 0-II |  |  |  |  |  |
| with complication | 335(39.37%) | 306(35.96%) | 210(24.68%) | 6.42 | 0.040 |
| without complication | 1,425(80.97%) | 1,107(78.34%) | 707(77.10%) |  |  |
| Stage III-IV |  |  |  |  |  |
| with complication | 101(19.09%) | 177(33.46%) | 251(47.45%) | 1.63 | 0.442 |
| without complication | 444(81.47%) | 752(80.95%) | 1,218(82.91%) |  |  |

^a^ The ANOVA test was used to calculate *p* value. The complication rates were only available for patients after surgery. Abbreviations: ESCC, esophageal squamous cell carcinoma

# References

1. Rice TW, Blackstone EH, Rusch VW. 7th edition of the AJCC Cancer Staging Manual: esophagus and esophagogastric junction. *Ann Surg Oncol* 2010; **17**(7): 1721-4.

2. The World Bank. Available at: https://data.worldbank.org/indicator/PA.NUS.PPP?skipRedirection=true&view=map&year=2023. Accessed July 31, 2024.
